# A Novel Signature Constructed by RNA-Binding Protein Coding Genes to Improve Overall Survival Prediction of Glioma Patients

**DOI:** 10.3389/fcell.2020.588368

**Published:** 2021-01-28

**Authors:** Zewei Tu, Lei Shu, Jingying Li, Lei Wu, Chuming Tao, Minhua Ye, Xingen Zhu, Kai Huang

**Affiliations:** ^1^Department of Neurosurgery, The Second Affiliated Hospital of Nanchang University, Nanchang, China; ^2^East China Institute of Digital Medical Engineering, Shangrao, China; ^3^Institute of Neuroscience, Nanchang University, Nanchang, China; ^4^Department of Comprehensive Intensive Care Unit, The Second Affiliated Hospital of Nanchang University, Nanchang, China

**Keywords:** glioma, RNA binding protein, overall survival, prognostic signature, biomarker

## Abstract

RNA binding proteins (RBPs) have been reported to be involved in cancer malignancy but related functions in glioma have been less studied. Herein, we screened 14 prognostic RBP genes and constructed a risk signature to predict the prognosis of glioma patients. Univariate Cox regression was used to identify overall survival (OS)-related RBP genes. Prognostic RBP genes were screened and used to establish the RBP-signature using the least absolute shrinkage and selection operator (Lasso) method in The Cancer Genome Atlas (TCGA) cohort. The 14 RBP genes signature showed robust and stable prognostic value in the TCGA training (*n* = 562) cohort and in three independent validation cohorts (Chinese Glioma Genome Atlas [CGGA]seq1, CGGAseq2, and GSE16011 datasets comprising 303, 619, and 250 glioma patients, respectively). Risk scores were calculated for each patient and high-risk gliomas were defined by the median risk score in each cohort. Survival analysis in subgroups of glioma patients showed that the RBP-signature retained its prognostic value in low-grade gliomas (LGGs) and glioblastomas (GBM)s. Univariate and multivariate Cox regression analysis in each dataset and the meta cohort revealed that the RBP-signature stratification could efficiently recognize high-risk gliomas [Hazard Ratio (HR):3.662, 95% confidence interval (CI): 3.187–4.208, *p* < 0.001] and was an independent prognostic factor for OS (HR:1.594, 95% CI: 1.244–2.043, *p* < 0.001). Biological process and KEGG pathway analysis revealed the RBP gene signature was associated with immune cell activation, the p53 signaling pathway, and the PI3K-Akt signaling pathway and so on. Moreover, a nomogram model was constructed for clinical application of the RBP-signature, which showed stable predictive ability. In summary, the RBP-signature could be a robust indicator for prognostic evaluation and identifying high-risk glioma patients.

## Introduction

Glioma, a type of intracranial tumor, is characterized by high invasiveness, obstinate recurrence and lethal malignancy (Jiang et al., 2016). According to the World Health Organization (WHO) classification, gliomas may be defined into four grades (grades I–IV). Diffuse low-grade (WHO grade II) and intermediate-grade (WHO grade III, anaplastic) gliomas are usually grouped as lower-grade gliomas (LGGs), while glioblastoma (GBM, WHO IV) is the deadliest type of glioma in human adults (Cancer Genome Atlas Research Network, 2008; Cancer Genome Atlas Research Network et al., 2015). Universal treatment of glioma involves surgical resection combined with chemotherapy or/and radiotherapy, but clean resection is extremely difficult to achieve due to its high invasiveness. Therefore, it is necessary to search for new effective biomarkers or targets to improve therapeutic effects and enhance our understanding of glioma treatment.

RNA-binding proteins (RBPs) are intracellular multifunctional proteins that combine with target RNAs to form ribonucleic protein complexes and regulate the processes of gene expression at the post-transcriptional level. These regulated processes include RNA splicing, polyadenylation, attenuation, editing, modification, and translation and are essential for maintaining cell metabolism and coordinating the maturity, transport, stability, and degradation of various RNAs (Gerstberger et al., 2014). Recent studies have found that aberrant expression of RBPs could affect cellular functions, leading to the occurrence and progression of various cancers, including gliomas. Further, growing evidence has associated RBP dysregulation to oncogenesis and cancer progression. For instance, ADDIN EN.CITE The RNA binding protein LIN28 could cooperate with WNT signaling to drive invasive intestinal and colorectal adenocarcinoma (Tu et al., 2015). HuR was identified as a RNA binding protein which acts as a crucial oncogenic driver and promote malignant peripheral nerve sheath tumors (MPNSTs) growth and metastasis (Palomo-Irigoyen et al., 2020). The YTH-domain family member 2 (YTHDF2) accelerates the degradation of EGFR mRNA by directly binding to the m6A modification site of the 3'-UTR of EGFR in HCC cells (Zhong et al., 2019).

RBPs are tightly associated with the initiation and progression of cancers, and a number of RBPs have been found contributing to the malignant phenotype of glioma, like the RNA binding protein IMP2 could maintain the glioblastoma stem cells by preventing let-7 target gene silencing (Janiszewska et al., 2012; Degrauwe et al., 2016), the oncogenic role of RNA-binding protein Musashi1 could be counteracted by miR-137 and inhibited by Luteolin (Yi et al., 2018; Velasco et al., 2019). Thus, we think exploring the roles and functions of latent RBPs in the initiation and development of glioma is needed and meaningful.

In present study, we screened prognostic RBPs of gliomas and constructed a 14 RBP gene-based risk signature to predict the OS of glioma patients in The Cancer Genome Atlas (TCGA) dataset. These results were validated in three external independent cohorts (Chinese Glioma Genome Atlas [CGGA]seq1, CGGAseq2, and GSE16011). Furthermore, associated biological processes and pathways were identified using differential expression of genes in low- and high-risk glioma subgroups, which might provide some clue to the potential function of these RBPs in glioma pathogenesis. Furthermore, a nomogram model integrating the risk signature, patient age, and the WHO grade was also constructed to predict the 1-, 3- and 5-year OS rates of glioma patients, to encourage the clinical application of our RBP-signature.

## Materials and Methods

### Data Acquisition

Four independent glioma cohorts were included in the present study. The transcriptome data of the TCGA training cohort was obtained from the website of Genomic Data Commons Data Portal (GDC; https://portal.gdc.cancer.gov/) and the corresponding clinical, pathological and molecular information was downloaded from the cBioPortal website (https://www.cbioportal.org/). As for the two validation RNA-seq cohorts CGGAseq1 and CGGAseq2, the related expression data and clinicopathological information were retrieved from the CGGA website (http://www.cgga.org.cn/). The microarray data of the GSE16011 validation cohort was obtained from the Gene Expression Omnibus (GEO) repository (https://www.ncbi.nlm.nih.gov/geo/), and the related clinical data was found in a previous study (Gravendeel et al., 2009). Similarly, the list of 1542 RBPs was based on that of a previous published study (Gerstberger et al., 2014).

### Patient Exclusion Criterion

We set the criteria for excluding glioma patients as follows: (a) glioma patients without OS information or OS < 30 days (to exclude statistical biases resulted for special short-lived cases); (b) patients without WHO grade information or expression data, and (c) patients with WHO grade I glioma. From the exclusion criterion, we obtained three RNA-seq cohorts (TCGA, CGGAseq1, and CGGAseq2) and one microarray cohort (GSE16011), which included 562, 303, 619, and 250 gliomas, respectively. The clinicopathological and molecular features of the glioma patients included in the present study are shown in Table 1.

**Table 1:** Clinicopathological and molecular information of glioma patients included in this study.

**Features**	**Total**	**TCGA cohort**	**CGGAseq1 cohort**	**CGGAseq2 cohort**	**GSE16011 cohort**
	**(*n* = 1,734)**	**(*n* = 562)**	**(*n* = 303)**	**(*n* = 619)**	**(*n* = 250)**
**Overall survival (years)**					
Median (range)	1.54 (0.08–20.68)	1.18 (0.08–17.34)	2.22 (0.09–11.41)	2.16 (0.11–11.98)	1.27 (0.08–20.68)
<5	1,428 (82.4%)	512 (91.1%)	201 (66.3%)	509 (82.2%)	206 (82.4%)
≥5	306 (17.6%)	50 (8.9%)	102 (33.7)	110 (69%)	44 (17.6%)
**Age (years)**					
Median (range)	45 (8–87)	47.5 (14–87)	42 (8–79)	43 (11–76)	51.5 (14–81)
<40	617 (35.6%)	197 (35.1%)	119 (39.3%)	235 (38.0%)	66 (26.4%)
≥40	1,115 (64.3%)	365 (64.9%)	184 (60.7%)	383 (61.9%)	183 (73.2%)
NA	2 (0.1%)	0 (0.0%)	0 (0.0%)	1 (0.2%)	1 (0.4%)
**Gender**					
Male	1,035 (59.7%)	326 (58.0%)	187 (61.7%)	356 (57.5%)	166 (66.4%)
Female	699 (40.3%)	236 (42.0%)	116 (38.3%)	263 (43.5%)	84 (33.6%)
**WHO grade**					
II	488 (28.1%)	196 (34.9%)	97 (32.0%)	173 (27.9%)	22 (8.8%)
III	598 (34.5%)	212 (37.7%)	73 (24.1%)	232 (37.5%)	81 (32.4%)
IV	648 (37.4%)	154 (27.4%)	133 (43.9%)	214 (34.6)	147 (58.8%)
**Histology**					
Astrocytoma	401 (23.1%)	150 (26.7%)	62 (20.5%)	163 (26.3%)	26 (10.4%)
Oligoastrocytoma	414 (23.9%)	102 (18.1%)	72 (23.8%)	213 (34.4%)	27 (10.8%)
Oligodendroglioma	271 (15.6%)	156 (27.8%)	36 (11.9%)	29 (4.7%)	50 (20.0%)
Glioblastoma	648 (37.4%)	154 (27.4%)	133 (43.9%)	214 (34.6%)	147 (58.8%)
**IDH mutation status**					
6 Mutant	823 (47.5%)	342 (60.9%)	165 (54.5%)	316 (51.1%)	NA
Wild	304 (17.5%)	212 (37.7%)	137 (45.2%)	258 (41.7%)	NA
NA	607 (35.0%)	8 (1.4%)	1 (0.3%)	45 (7.3%)	NA
**1p/19q codeletion status**					
Non-codeletion	1,080 (62.3%)	418 (74.4%)	235 (77.6%)	427 (69.0%)	NA
Codeletion	329 (19.0%)	138 (24.6%)	63 (20.8%)	128 (20.7%)	NA
NA	325 (18.7%)	6 (1.1%)	5 (1.7%)	64 (10.3%)	NA

### Data Processing

For the three RNA-seq cohorts, the Fragments Per Kilobase of transcript per Million (FPKM) data values were downloaded and were transformed to Transcripts Per Kilobase Million (TPM) values using an algorithm described in previous studies(Li et al., 2010; Wagner et al., 2012). The TPM values were used in the subsequent analysis. For the microarray data relative to the GSE16011 cohort, the raw data of “CEL” files were used to perform background adjustments and quantile normalization using a robust multiarray averaging method (RAM) with the R packages “affy” (Gautier et al., 2004) and “simpleaffy” (Wilson and Miller, 2005).

### Construction of the RBP-Signature

As the training cohort, TCGA dataset was used to conduct univariate Cox regression analysis to screen for OS-related RBP genes in gliomas. A total of 662 OS-related RBP genes were identified (*p* < 0.05). Next, Lasso Cox regression method, a recommended method for regression of high-dimensional microarray data, was used to fit the OS-related RBPs data and construct a risk signature based on the selected 662 RBP genes. A formula was generated to calculate risk scores for glioma patients with the relative expression value of RBP genes and respective coefficients. The formula obtained was the following:

(1) $$\begin{array}{l} risk\;score=\overset{n}{\underset{i=1}{\sum }}Coef_{i}*x_{i} \end{array}$$

in which the *Coef*_*i*_ is the coefficient of each RBP genes, and *x*_*i*_ is the TPM expression value or RMA normalized value of each selected RBP gene in each cohort.

### Biological Process and Pathway Analysis

Differential expression analysis was conducted between low- and high-risk subgroups using the “limma” (Ritchie et al., 2015) package to screen differentially expressed genes (DEGs) in the TCGA cohort. The TPM expression value was used for differential expression analysis and genes with log2 (fold change) >1 and *p* < 0.05 were defined as DEGs between low- and high- risk subgroups. A total 3,672 genes were identified and were used to perform gene ontology biological processes (GO-BP) analysis and Kyoto Encyclopedia of Genes and Genomes (KEGG) analysis using the “clusterProfiler” package (Yu et al., 2012).

### Construction and Validation of the Nomogram Model

The R package “rms” was used to construct a nomogram model and for its validation. By balancing the prognostic value and accessibility of including predictors, the patient's age, WHO grade and risk score were included in our nomogram model, based on the results of multivariate Cox regression. Due to the existing batch effects among the four cohorts (3 RNA-seq cohorts and 1 microarray cohort), glioma patients were divided into low- and high-risk, as a dichotomous variable, by the median risk score in each cohort. Age and WHO grade were used as continuous variables in the nomogram model. Calibration plots were performed using the “calibrate” function of the “rms” package.

### Cell Line and Cell Culture

We purchased astrocytoma cell line SW1088 and GBM cell line U251 and LN229 from the Chinese Academia Sinica Cell Repository (Shanghai, China). Glioma cells were cultured using Dulbecco's modified Eagle's medium (DMEM; Gibco, USA) containing 10% fetal bovine serum (FBS) (Gibco, USA) and 1% penicillin and streptomycin (Gibco, USA). All cells were cultured at 37°C in filtered air with 100% humidity and 5% CO_2_.

### Western Blotting and Antibody

Cultured cells were rinsed with phosphate buffered saline (PBS) and lysed for 15 min by ice-cold cell lysis buffer (Solarbio, China) on the ice. Then lysed cell samples were centrifuged at 12,000g for 15 min at 4°C, and the supernatant liquid of each sample was gathered. Bicinchoninic acid assay (BCA) kit (KeyGEN Biotech, China) was used for protein concentration measurement. Equivalent proteins of each sample were added in each lane and separated by 10% sodium dodecyl sulfate (SDS)–polyacrylamide gel electrophoresis. Subsequently, proteins were transferred onto polyvinylidene difluoride-membranes (PVDF membranes, Millipore, MA, USA) and membranes were blocked in 10% bull serum albumin (BSA) at room-temperature for 1 h. Blocked PVDF membranes were incubated with primary antibodies overnight at 4°C. The antibodies used in present study were rabbit anti-GNL1 (1:500, 14078-1-AP, Proteintech, China), rabbit anti-RDM1 (1:500, 20156-1-AP, Proteintech), rabbit anti-FBXO17 (1:500, 12844-1-AP, Proteintech), rabbit anti-SPATS2L (1:500, 16938-1-AP, Proteintech, China), rabbit anti-GAPDH (1:5000, 10494-1-AP, Proteintech) and rabbit anti-bTubulin (1:1,000, 10068-1-AP, Proteintech). The membranes were then incubated with horseradish peroxidase (HRP)-conjugated affinipure goat anti-rabbit IgG (1:2,000, SA00001-2, proteintech) for 2 h at room- temperature. The gray values of the protein bands were calculated using the Image J software (National Institutes of Health, USA) and normalized to the GAPDH or b-Tubulin signal.

### Statistical Analysis

The two-sided log-rank test was used to compare the clinical outcomes between low- and high-risk subgroups or low- and high-expression of low- and high-expression subgroups of RBP genes using Kaplan-Meier curve analysis. Time-dependent receiver operating characteristic (ROC) curves were obtained to assess the OS predictive ability of the risk score. The prognostic role of RBP genes were evaluated by univariate Cox regression analysis. The Kruskal test was used to compared the expression levels of RBP genes in gliomas with different histological types and WHO grades. Regarding OS information, univariate and multivariate Cox regression analyses were used to assess the independent prognostic power of the RBP-signature. All statistical analysis in present study were conducted based on the R programming (version 3.6.1, https://www.r-project.org/) and SPSS Statistics version 25 (https://www.ibm.com/products/software).

## Results

### Development of the Prognostic RBP-Signature of Glioma Patients

**Figure 1:**
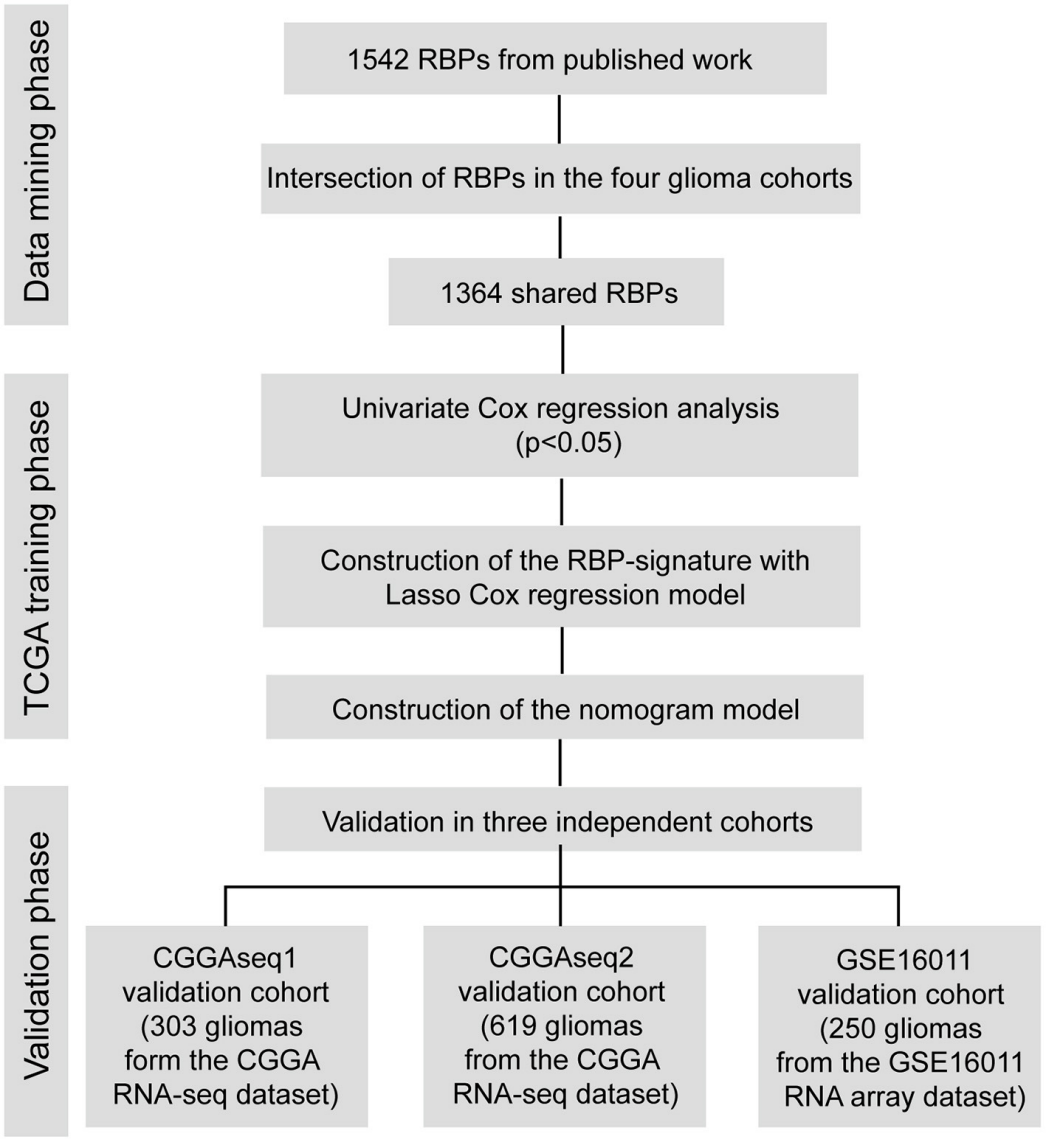
Flow chart of this study.

**Figure 2:**
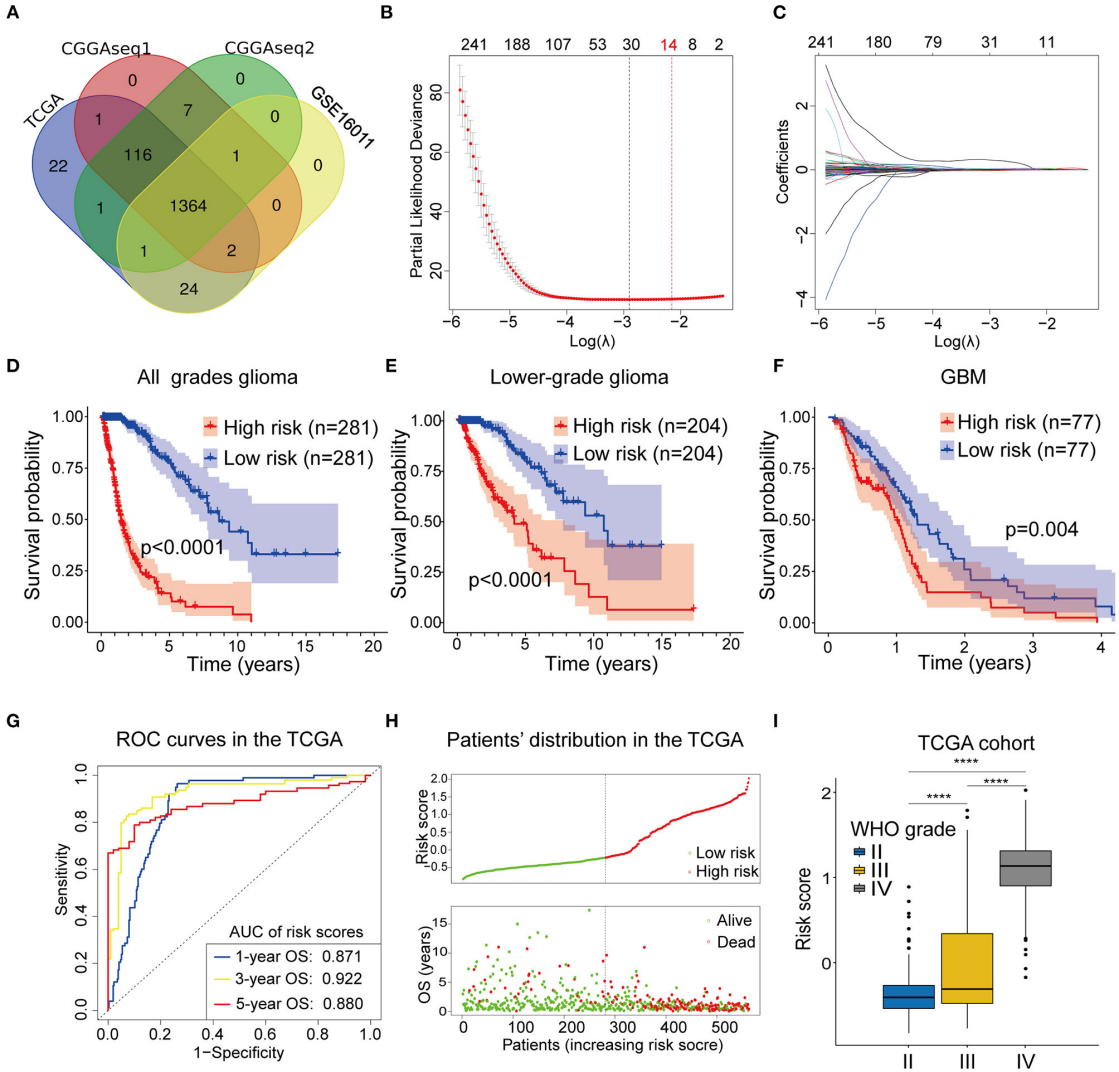
**(A)** The Venn plot showing the 1364 RBP genes identified in all the four cohorts. **(B,C)** Least absolute shrinkage and selection operator (Lasso) regression was performed to calculate **(B)** the minimum criteria and **(C)** coefficients. **(D–F)** The RBP-signature stratifies **(D)** glioma patients, **(E)** LGGs, and **(F)** GBMs into two subgroups with significantly different clinical outcomes. **(G)** Receiver operating characteristic (ROC) curves showing the 1-, 3-, and 5-year OS predictive efficiency of the RBP-signature. **(H)** The patient distribution plot showing that patients with higher risk scores are associated with shorter OS. **(I)** The box plot indicating that WHO grades are positively associated with risk scores.

A flow chart describing our study process is shown in Figure 1. In the beginning, we extracted the RBPs expression matrix form the four cohorts and intersected the RBPs detected in all cohorts. A total 1,364 RBPs were detected in all cohorts (Figure 2A). Using the univariate Cox regression analysis, we selected 662 prognostic RBP genes, which were correlated with the OS of glioma patients (*p* < 0.05), from the shared 1,364 RBPs in the TCGA training cohort. These RBPs were used as the candidate prognostic genes for constructing the RBP-signature for glioma patients.

Next, we performed the Lasso Cox regression analysis to construct the RBP-signature in the TCGA training cohort (Figures 2B,C) and the 14-RBP signature was built for predicting the OS of glioma patients. Meanwhile, risk scores were calculated for each glioma patient based on their 14 RBP genes expression value and related coefficients. The risk score = (ANG^*^0.00704479286224555) + (APOBEC3F^*^0.0166487349839528) + (CARHSP1^*^1.85993728316624e-05) + (CTIF^*^-0.00289009741903594) + (FBXO17^*^0.0212696099048724) + (GNL1^*^-0.0077699903265289) + (ISG20^*^0.0243466438725418) + (KHNYN^*^0.00375904579150528) + (LSM12^*^0.00192073111674367) + (PABPC3^*^0.0467898021506214) + (PNRC2^*^0.000388662732954364) + (RDM1^*^0.0424311619487984) + (SPATS2L^*^0.00898980677256576) + (TTF2^*^0.0063890105865751).

The formula was also used to calculated risk scores for the glioma patients in the three validation cohorts.

To judge the predictive ability of this RBP risk model, we classified 562 glioma patients into low- and high-subgroups based on the median value of the risk scores. Survival analysis revealed that high-risk glioma patients were significantly associated with worse clinical outcomes (Figure 2D, *p* < 0.0001). Furthermore, survival analysis was also performed in LGGs (Figure 2E, *p* < 0.0001) and GBMs (Figure 2F, *p* = 0.004) patients in the TCGA training cohort, using the median value of risk scores as the cut off value because all GBMs in the TCGA cohort were categorized as high-risk gliomas.

To determine the predictive ability of the RBP-signature, we conducted a time-dependent ROC analysis using the risk scores in the TCGA cohort. The results showed that the area under the curves (AUC) of the 1-, 3-, 5-year OS predicted using the ROC curves were 0.871, 0.922, and 0.870, respectively (Figure 2G). The patient distribution plot indicated that high-risk glioma patients were associated with lower OS (Figure 2H) and the patients' risk scores were positively related to their WHO grades (Figure 2I) in the TCGA training cohort. These results indicated that our RBP-signature possessed a predictive ability with high accuracy for patients with gliomas.

### Validation of the Prognostic Value of RBP-Signature

**Figure 3:**
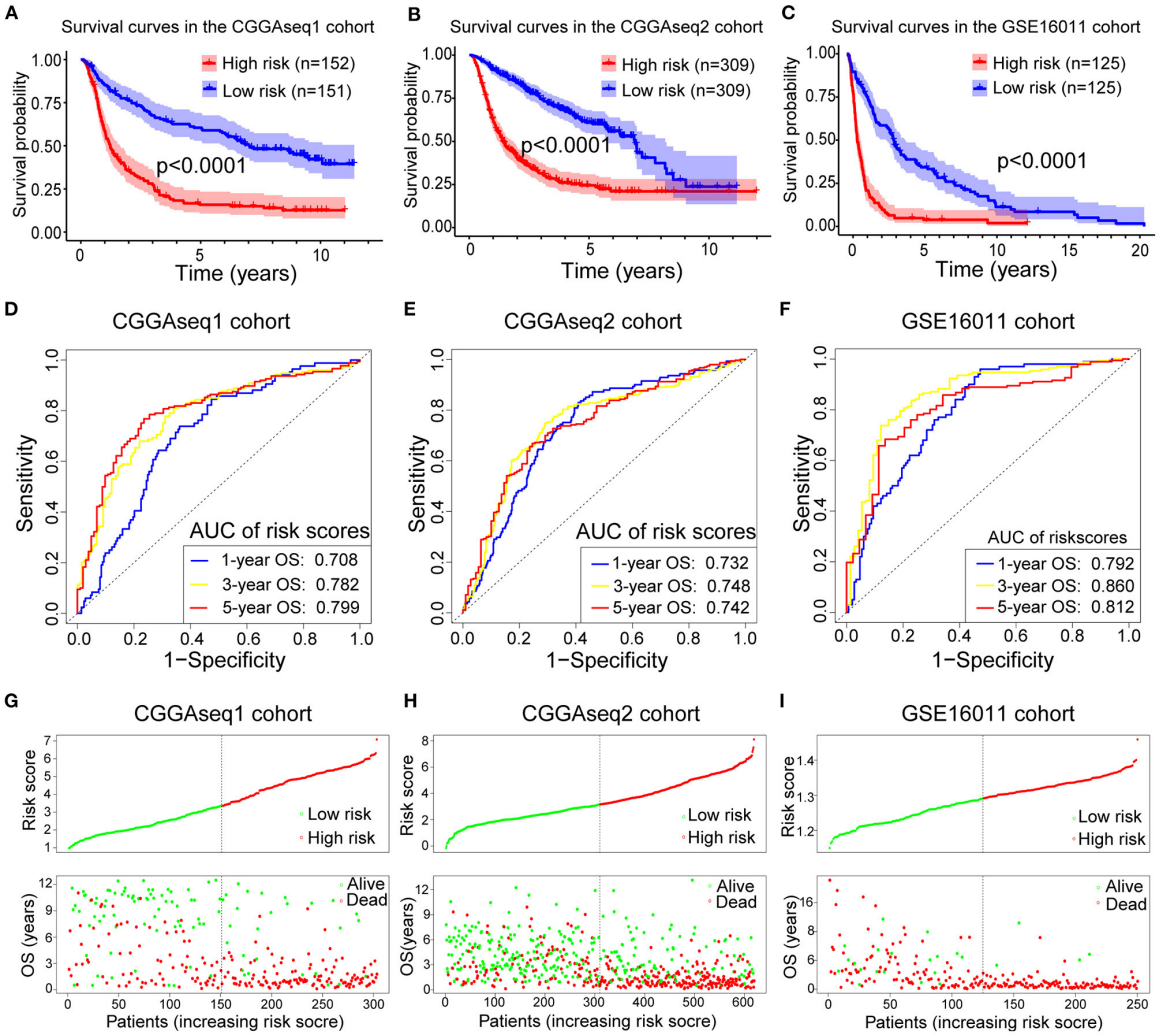
**(A–C)** Survival curves showing how the RBP-signature stratifies glioma patients into two subgroups with significantly distinct clinical outcomes in the three validation cohorts. **(D–F)** Receiver operating characteristic (ROC) curves showing the 1-, 3-, and 5-year OS predictive efficiency of the RBP-signature in the three validation cohorts. **(G–I)** Patient distribution plots showing patients with higher risk scores are associated with shorter OS in the three validation cohorts.

To analyze the robustness and stability of the RBP-signature, we performed a survival analysis and ROC analysis using the other three cohorts (CGGAseq1, CGGAseq2, and GSE16011). Using the same formula, risk scores were calculated for patients in the validation cohorts dividing them into low- and high- subgroups based on the median value in each cohort. According to our validation results, glioma patients in the high-risk subgroups had worse OS and survival rates compared to low-risk glioma patients in the three validation cohorts (Figures 3A–C, *p* < 0.0001 for all three cohorts). Furthermore, the high-risk subgroups in the three validation cohorts had significantly worse OS compared with the low-risk subgroups in the LGGs (Supplementary Figures 1A–C, *p* < 0.001) and similar results were also obtained for the GBMs (Supplementary Figures 1D–F) with *p*-values of 0.271 (CGGAseq1), 0.019 (CGGAseq2), and 0.0049 (GSE16011), respectively. The results showed that the RBP-signature retained its prognostic value in LGG and GBM patients.

The ROC analysis in the validation cohorts showed that the RBP-signature had a stable and robust OS-predictive ability in glioma patients. The AUC of predicting 1-, 3-, and 5-year OS in the three validation cohorts were 0.708, 0.782, and 0.799 (CGGAseq1 cohort), 0.732, 0.748, and 0.742 (CGGAseq2 cohort), and 0.792, 0.860, and 0.812 (GSE16011 cohorts), respectively (Figures 3D–F). The patient distribution plots in the other three validation cohorts also showed that glioma patients with higher risk scores were associated with shorter OS (Figures 3G–I).

### The Prognostic Value and Expression Levels of the 14 RBPs in Gliomas

**Figure 4:**
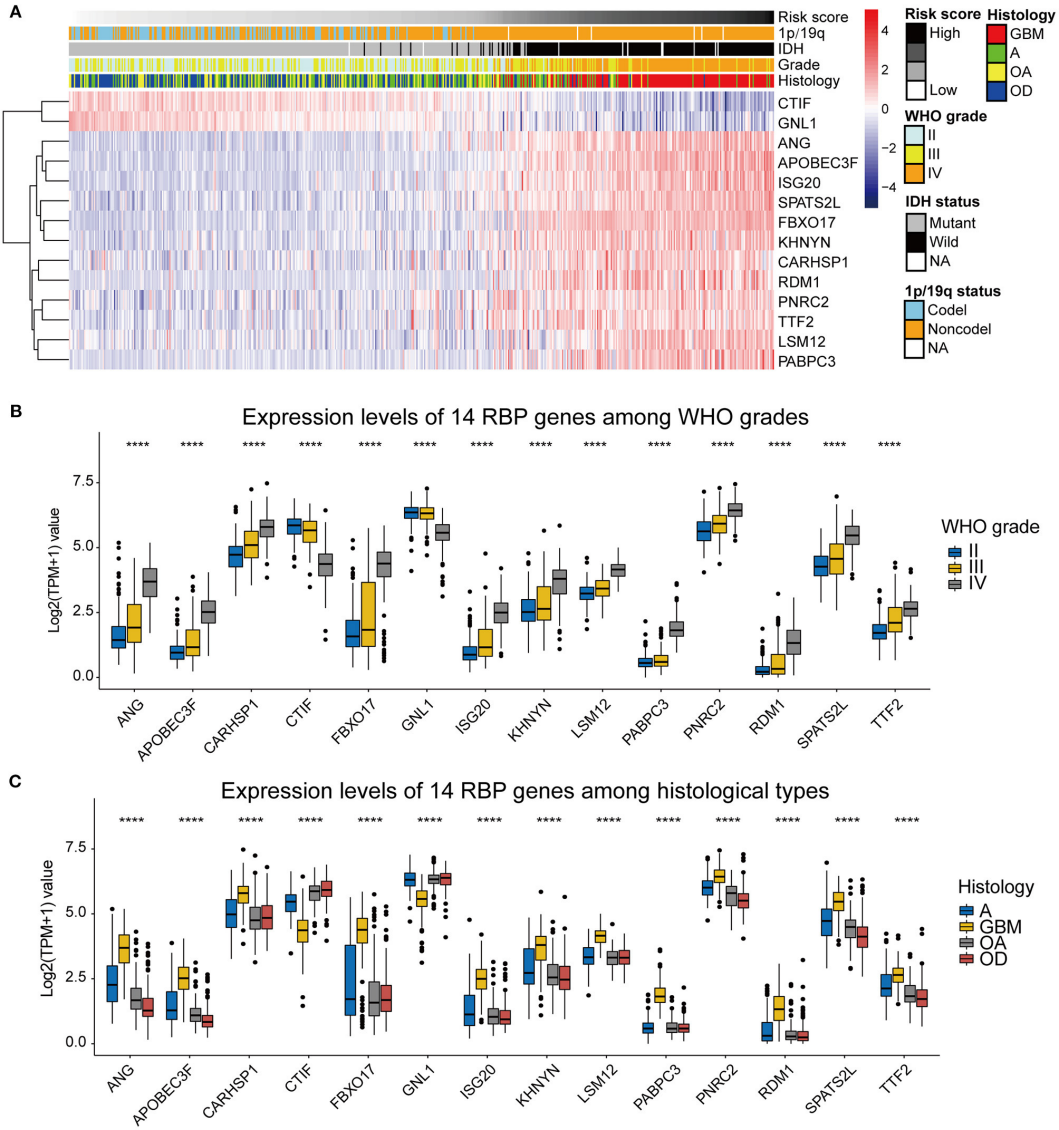
**(A)** The heatmap showing the correlation between the expression levels of the 14 RBP genes and the clinicopathological features including the risk score, 1p/19q codeletion status, IDH mutational status, WHO grade, and histological types. **(B)** Expression levels of the 14 RBP genes among gliomas with different WHO grades. **(C)** Expression levels of the 14 RBP genes among gliomas with different histological types. GBM, glioblastoma; A, astrocytoma; OA, oligoastrocytoma; OD, oligodendroglioma. *p < 0.05, **p < 0.01, ***p < 0.001, ****p < 0.0001.

To assess the roles of each of the 14 RBPs in gliomas, we designed a heatmap to visualize the associations between the expression levels of the 14 RBPs and common molecular and pathological features of gliomas, including the risk score, IDH mutational status, 1p/19q codeletion status, WHO grade, and histological type (Figure 4A). In addition, the expression levels of the 14 RBPs for each of the WHO grades and histological types were also visualized using box plots (Figures 4B,C). The results showed that CTIF and GNL1 mRNA expression was reduced and that of ANG, APOBEC3F, CARHSP1, FBXO17, ISG20, KHNYN, LSM12, PABPC3, PNRC2, RDM1, SPATS2L, and TTF2 mRNA increased in WHO grade IV (GBM) compared with those of LGGs. To evaluate the prognostic roles of these RBPs in glioma patients, univariate Cox regression (Table 2) and a two-sided log-rank test (Supplementary Figure 1) were performed. The results showed that CTIF and GNL1 were potential protective genes and ANG, APOBEC3F, CARHSP1, FBXO17, ISG20, KHNYN, LSM12, PABPC3, PNRC2, RDM1, SPATS2L, and TTF2 might act as risk factors in glioma patients.

**Table 2:** Coeffients and univariate Cox regression analysis of 14 RBP genes.

**Gene**	**Coeffients**	**Description**	**HR**	**HR.95L**	**HR.95H**	***p*-value**
CTIF	−0.002890097	CBP80/20-dependent translation initiation factor	0.953	0.944	0.961	<0.001
GNL1	−0.00776999	Guanine nucleotide binding protein-like 1	0.965	0.959	0.971	<0.001
ANG	0.007044793	Angiogenin, ribonuclease, RNase A family, 5	1.083	1.068	1.099	<0.001
APOBEC3F	0.016648735	Apolipoprotein B mRNA editing enzyme, catalytic polypeptide-like 3F	1.307	1.256	1.360	<0.001
CARHSP1	0.0000186	Calcium regulated heat stable protein 1	1.024	1.019	1.029	<0.001
FBXO17	0.02126961	F-box protein 17	1.071	1.060	1.082	<0.001
ISG20	0.024346644	Interferon stimulated exonuclease gene 20kDa	1.173	1.143	1.203	<0.001
KHNYN	0.003759046	KH and NYN domain containing	1.077	1.064	1.091	<0.001
LSM12	0.001920731	LSM12 homolog	1.174	1.142	1.207	<0.001
PABPC3	0.046789802	poly(A) binding protein, cytoplasmic 3	1.371	1.299	1.446	<0.001
PNRC2	0.000388663	Proline-rich nuclear receptor coactivator 2	1.026	1.020	1.031	<0.001
RDM1	0.042431162	RAD52 motif 1 [Source:HGNC Symbol;Acc:19950]	1.552	1.439	1.674	<0.001
SPATS2L	0.008989807	Spermatogenesis associated, serine-rich 2-like	1.042	1.035	1.048	<0.001
TTF2	0.006389011	Transcription termination factor, RNA polymerase II	1.179	1.138	1.221	<0.001

Furthermore, we also measured the protein levels of four selected RBPs (GNL1, SPATS2L, RDM1, and FBXO17) in three glioma cell lines, one astrocytoma cell line (SW1088) and two glioblastoma cell lines (U251 and LN229). Western blotting results showed that protein level of GNL1 was significantly lower in U251 and LN229 cells compared with SW1088 cells (Supplementary Figure 2A), while SPATS2L, RDM1, and FBXO17 protein expression levels were significantly higher in U251 and LN229 compared with SW1088 cells (Supplementary Figures 2B–D). These results were consistent with the conclusions in the datasets analysis.

### Identifying the RBP-Signature Related Biological Processes and Pathways

**Figure 5:**
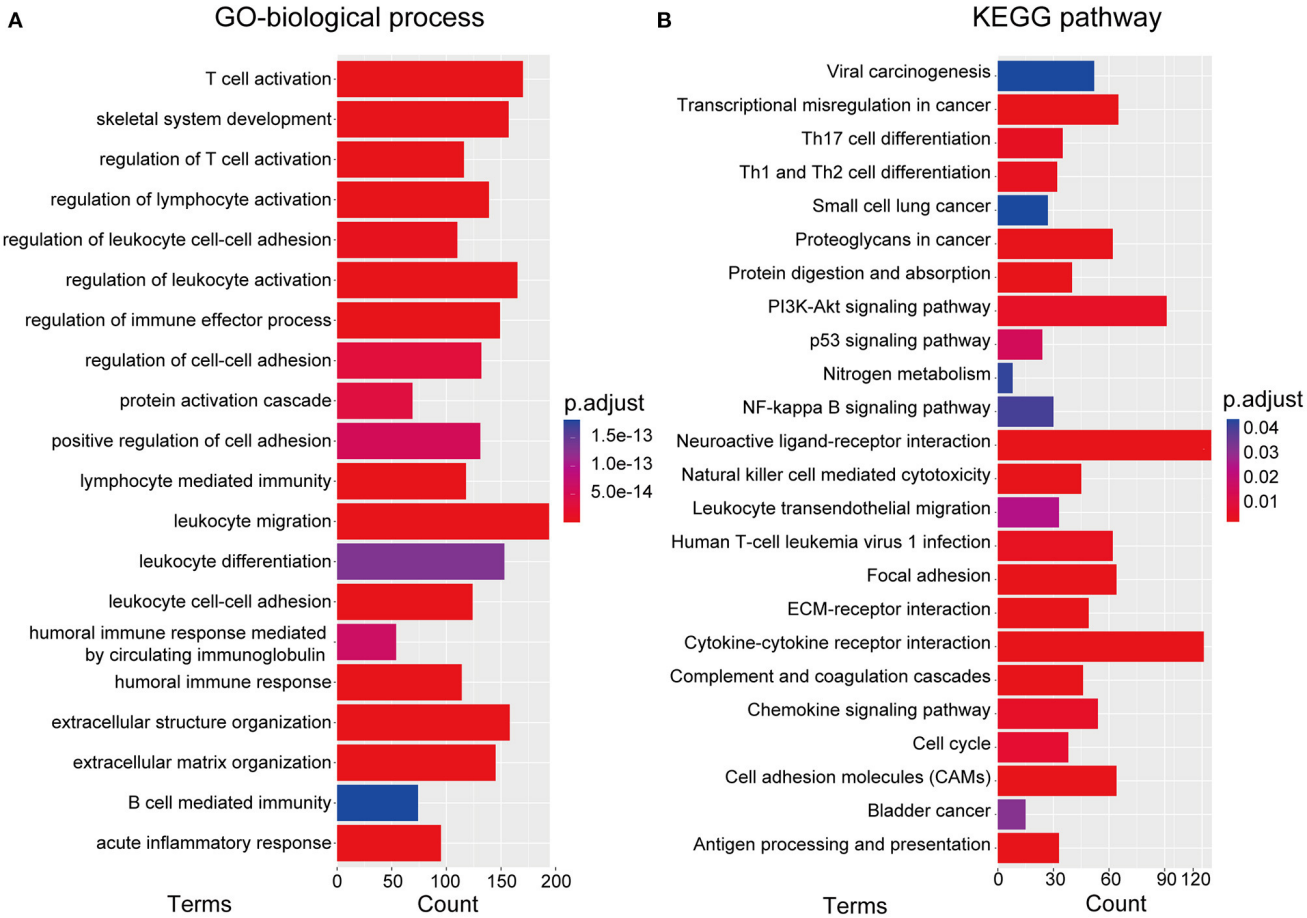
**(A)** Gene ontology biological process (GO-BP) analysis. **(B)** Kyoto Encyclopedia of Genes and Genomes (KEGG) pathway analysis.

To investigate the underlying biological processes and pathways associated with the RBP-signature, we performed differential expression analysis between the low- and high-risk glioma patients in the TCGA cohort. A total of 3,672 DEGs were identified with the standard of |log2(fold change)| >1 and *p*-value < 0.05 and were used to perform GO-BP and KEGG pathway analysis using the R package “clusterProfiler.” We found that the DEGs were mainly enriched in immune cell-related biological processes such as T cell activation, regulation of lymphocyte activation, regulation of leukocyte activation, regulation of immune effector process, lymphocyte mediated immunity, leukocyte migration, leukocyte differentiation, leukocyte cell-cell adhesion, and B cell mediated immunity (Figure 5A). Cancer-associated pathways were also enriched in the high-risk gliomas identified by the RBP-signature, including transcriptional dysregulation in cancer, proteoglycans in cancer, the PI3K-Akt signaling pathway, the p53 signaling pathway, the NF-kappa B signaling pathway, focal adhesion, ECM-receptor interaction, and the chemokine signaling pathway (Figure 5B).

### Independent Prognostic Value of the RBP-Signature Stratification

**Figure 6:**
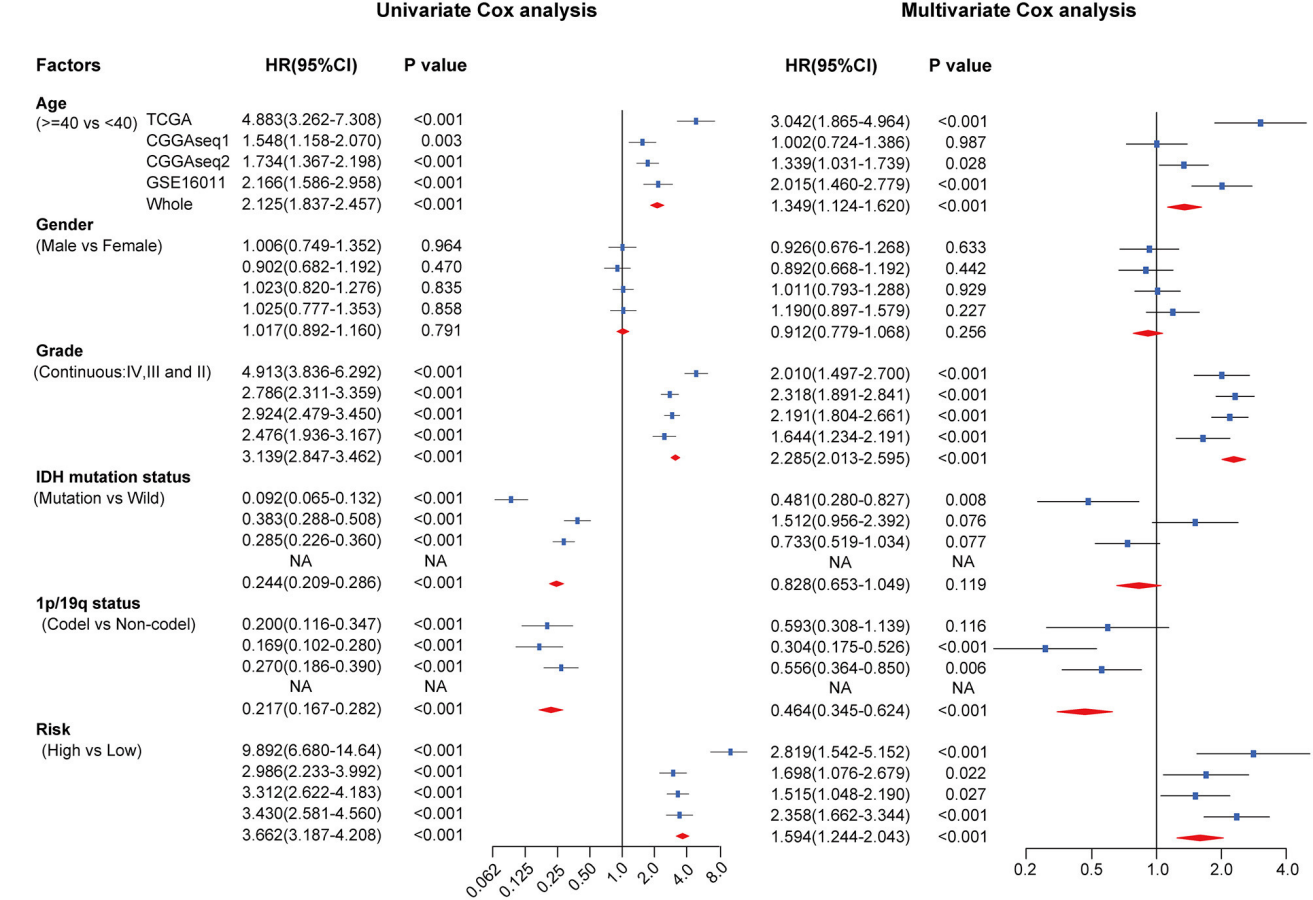
Univariate and multivariate Cox analysis of clinicopathological and molecular features (including age, sex, WHO grade, IDH mutational status, 1p/19q codeletion status and RBP-signature) in each cohort and in the meta cohort.

To assess the independent prognostic value of the RBP-signature, we carried out univariate and multivariate Cox analysis in the TCGA training cohort and in the three external validation cohorts. Risk scores were dichotomized as low- and high-risk according to the median value of risk scores in each cohort to evaluate the prognostic value of RBP-risk stratification. The results showed that the RBP-risk stratification method was not only an OS-related prognostic factor in gliomas, but it was confirmed as an independent prognostic factor of glioma patients in each independent cohort and in the combined cohort (Figure 6). The RBP-signature could successfully identify high-risk gliomas in each cohort and in the meta cohort (HR: 3.662, 95% CI: 3.187–4.208, *p* < 0.001) and was an independent prognostic factor (HR: 1.594, 95% CI: 1.244–2.043, *p* < 0.001). These data indicated that the RBP-signature may find clinical application as a promising predictor of OS and to predict the prognosis of glioma patients.

### Establishment and Validation of a Nomogram Model Based on the RBP-Signature Stratification

**Figure 7:**
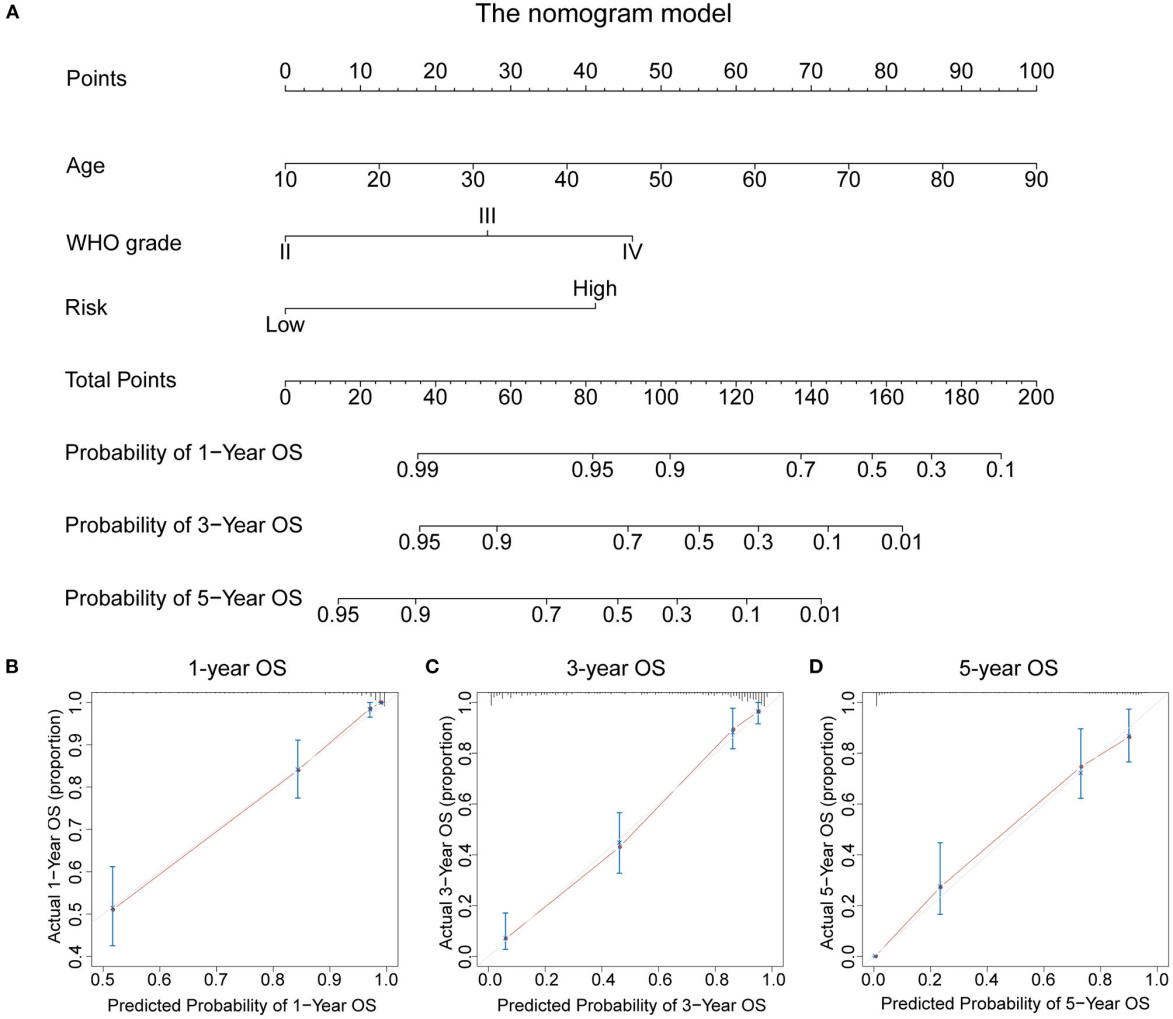
**(A)** The nomogram constructed using age, WHO grade, and the RBP-signature in the TCGA cohort. **(B–D)** Calibration plots indicating how the nomogram effectively predicts the 1-, 3-, and 5-year OS of glioma patients in the TCGA cohort.

To evaluate the potential clinical application of the RBP-signature, we constructed a nomogram model based on age, WHO grade, and the RBP-signature using multivariate Cox regression in the TCGA training cohort (Figure 7A). Age and WHO grade were included in the nomogram model for their independent prognostic ability and clinical accessibility. The calibration curves revealed that the nomogram had high predictive accuracy in forecasting the 1-, 3-, and 5-year OS of glioma patients in the TCGA cohort (Figures 7B–D), and it also showed good accuracy of OS prediction in the other three validation cohorts (Supplementary Figure 3). The C-index was also calculated to evaluate the discriminative ability of our nomogram model and it performed well (0.856 for the TCGA training cohort and 0.723 for the CGGAseq1, 0.737 for the CGGAseq2, and 0.732 for the GSE16011 validation cohorts). These results indicated that the RBP-signature-based nomogram model might represent a promising prognostic model for the evaluation of clinical prognosis.

## Discussion

Numerous predictive models for prognosis have emerged for cancer patients, which have also been followed by some avoidable limitations. First, many studies have used Cox proportional hazards regression analysis to generate a mRNA-based risk model. However, the Cox proportional hazards regression model was an effective approach to create a prognostic risk signature, but it is not the most appropriate choice in the face of high-dimensional RNA sequence or microarray data given the issue of overfitting of data. Secondly, redundant genes could be a barrier to clinical application. Some previously reported risk models contained too many genes or non-coding RNAs, and we thought that a risk signature constructed by moderate genes could be more feasible for clinical use. Finally, biases have been reported when a risk model is constructed based on a cohort of a limited number of patients a training cohort with fewer samples may lead a labile model which may not be successfully validated in other independent cohorts.

Growing evidence has indicated that dysregulation of RBPs occurs in different types of cancers but only a few RBPs have been verified to play a crucial role in oncogenesis and cancer progression. The roles of RBPs in glioma require further clarification. In present study, we identified 14 prognostic RBPs and constructed an RBP-signature to improve the predictive ability of OS time of glioma patients in the TCGA cohort (*n* = 562), which was assessed in four additional independent glioma cohorts and confirmed its robust and stable prognostic value.

Since our RBP-signature with 14 RBP genes was a good predictor of the survival risk of glioma patients, it is essential for us to investigate the underlying mechanisms involved. Potential relative biological processes and pathways of the RBP-signature have been suggested in our study, and the results showed that genes correlated with the RBP-signature were enriched in immune cell activities, cell adhesion or extracellular matrix organization, and in cancer-associated pathways. T cell, lymphocyte cell, and B cell immunity were found to be associated with our RBP-signature, which means patients with different risk scores have distinct immune responses or present a specific immune cell microenvironment within gliomas. Association with the biological processes of cell-adhesion and extracellular matrix organization might indicate that gliomas with higher risk scores possess higher invasive ability for infiltrating to peritumoral tissues. The p53 signaling pathway, PI3K-Akt signaling pathway and NF-kappa B signaling pathway, which were highly dysregulated in cancers, were identified analysis associated with the RBP-signature in the KEGG pathway analysis. These data might provide some clues for the underlying mechanisms that distinguish different OS of low- and high- risk glioma patients defined by the RBP-signature.

The biological functions of the 14 RBP genes have been moderately investigated but only a few have been reported in glioma. GNL1, or guanine nucleotide binding protein-like 1, belongs to the HSR1_MMR1 subfamily of nucleolar GTPases, and may be involved in the acceleration of the cell cycle and cell proliferation via enhancing the phosphorylation of retinoblastoma protein (Boddapati et al., 2012). The protein encoded by ANG, angiogenin, a member of the RNase A superfamily, is also a powerful promoter of new blood vessel formation. APOBEC3F, a member of the cytidine deaminase gene family, is thought to play a role in the cell cycle or growth control, and overexpression of APOBEC3F is also related to poor recurrence-free survival of HBV-related hepatocellular carcinoma (Yang et al., 2015). ISG20 is an interferon-induced exoribonuclease and usually acts on single-stranded RNA and exerts little effect on single-stranded DNA (Horio et al., 2004) and it may be induced by thyroid hormone and promotes angiogenesis in liver cancer (Lin et al., 2018). FBXO17 could promote liver cancer progression via the Wnt/β-catenin pathway and accelerate lung adenocarcinoma cell proliferation by activating the Akt pathway (Suber et al., 2018; Liu et al., 2019). RDM1 is reported to be involved in the cell response to cisplatin (Hamimes et al., 2005) and acts as an oncogene in several cancers; however, loss of RDM1 could promote liver cancer progression by the Ras/Raf/ERK and p53 pathways (Chen et al., 2020). PNRC2 encodes a coactivator which interacts with nuclear receptors using a proline-rich sequence (Zhou and Chen, 2001), and it may be targeted by mir-23a-3p to further promote the progression of renal cell carcinoma (Quan et al., 2019). Evidences above indicated that these screened RBPs might play a vital role in cancer and attention should be given to their role in glioma.

Despite the strengths of our study, there are also limitations to be addressed. Firstly, the data analyzed in our study derived from open-access datasets and our results were validated in retrospective rather than prospective cohorts. Secondly, some important molecular features in the GSE16011 cohort, including the 1p/19q co-deletion and the IDH mutational status, were missing; thus, they could not be analyzed in our study. Finally, more experimental studies should be performed to investigate the functional role of the 14 RBP genes included in the signature and to explore the correlation between the RBP-signature and prognosis of glioma patients.

In conclusion, this study successfully constructed an RBP-based risk signature that can efficiently predict the OS of glioma patients. The RBP-signature is a helpful complement to the prognostic indicators of glioma patients. However, our retrospective study requires further validation in other prospective glioma cohorts.

## Data Availability Statement

Publicly available datasets were analyzed in this study. This data can be found here: The data analyzed in this study can be acquired in the TCGA (http://cancergenome.nih.gov/), CGGA (http://www.cgga.org.cn/), and GEO (https://www.ncbi.nlm.nih.gov/geo/) websites.

## Author Contributions

XZ and KH constructed this study. ZT, LS, and JL performed the data analysis, figures plotted, and writing. LW, MY, and CT were responsible for the data acquisition and critical reading of the manuscript. All authors contributed to the article and approved the submitted version.

## Conflict of Interest

The authors declare that the research was conducted in the absence of any commercial or financial relationships that could be construed as a potential conflict of interest.
