# Supplementary figures and images for: A Novel Signature Constructed by RNA-Binding Protein Coding Genes to Improve Overall Survival Prediction of Glioma Patients
Source: Front Cell Dev Biol. 2021 Jan 28;8:588368. doi: 10.3389/fcell.2020.588368 (PMC7901892; doi:10.3389/fcell.2020.588368)

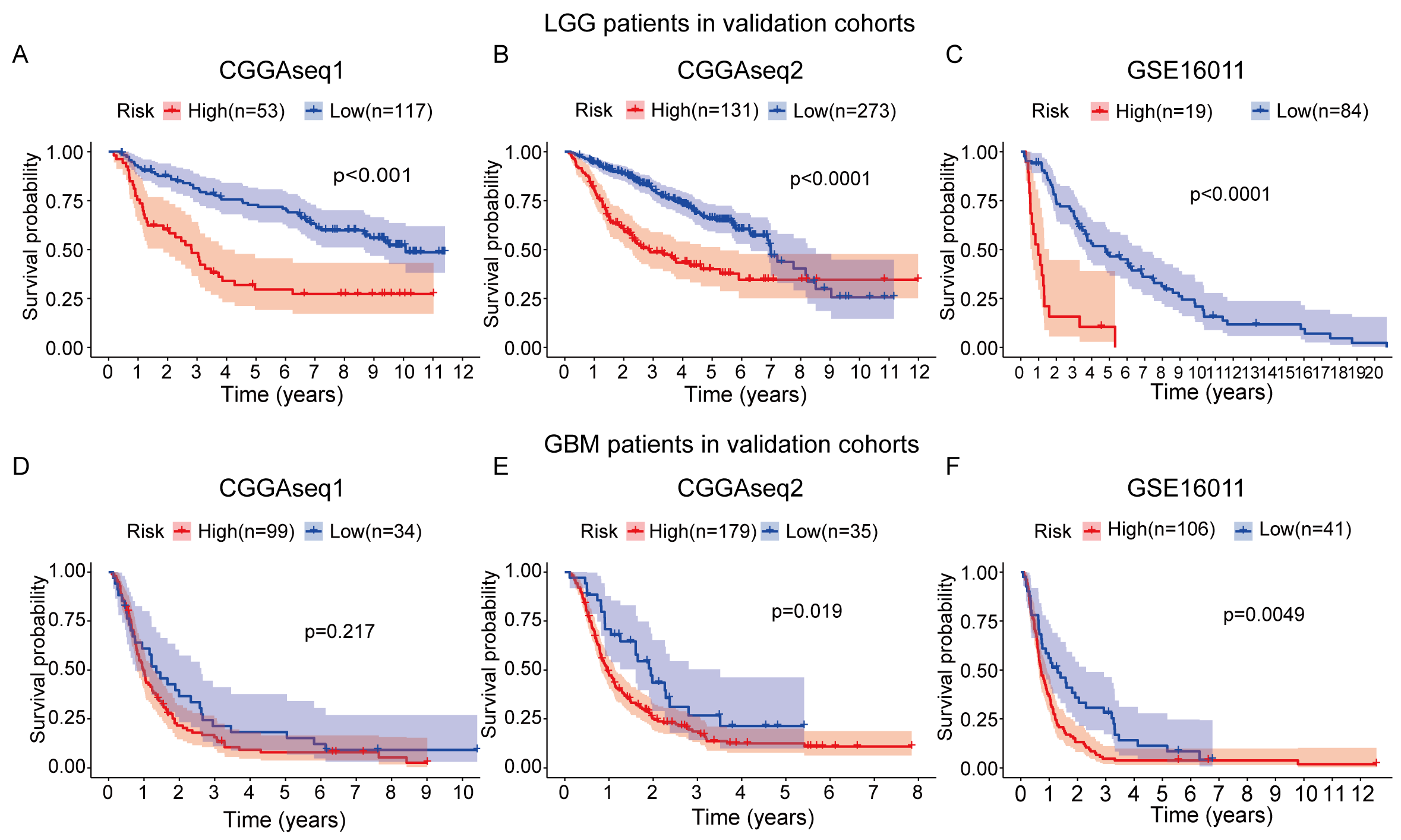

Supplement: Supplementary file 1 [file Image_1.TIF]

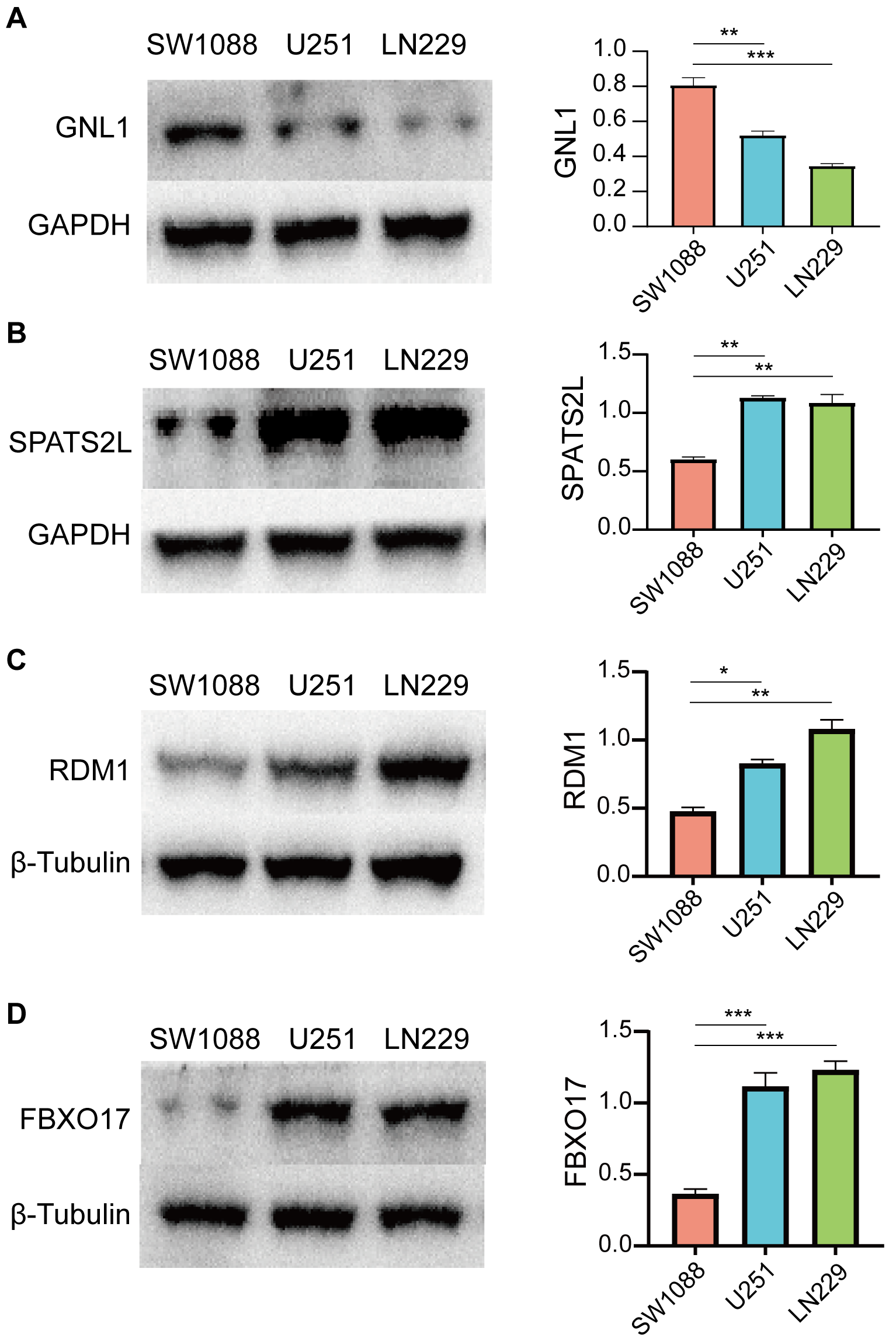

Supplement: Supplementary file 2 [file Image_2.TIF]

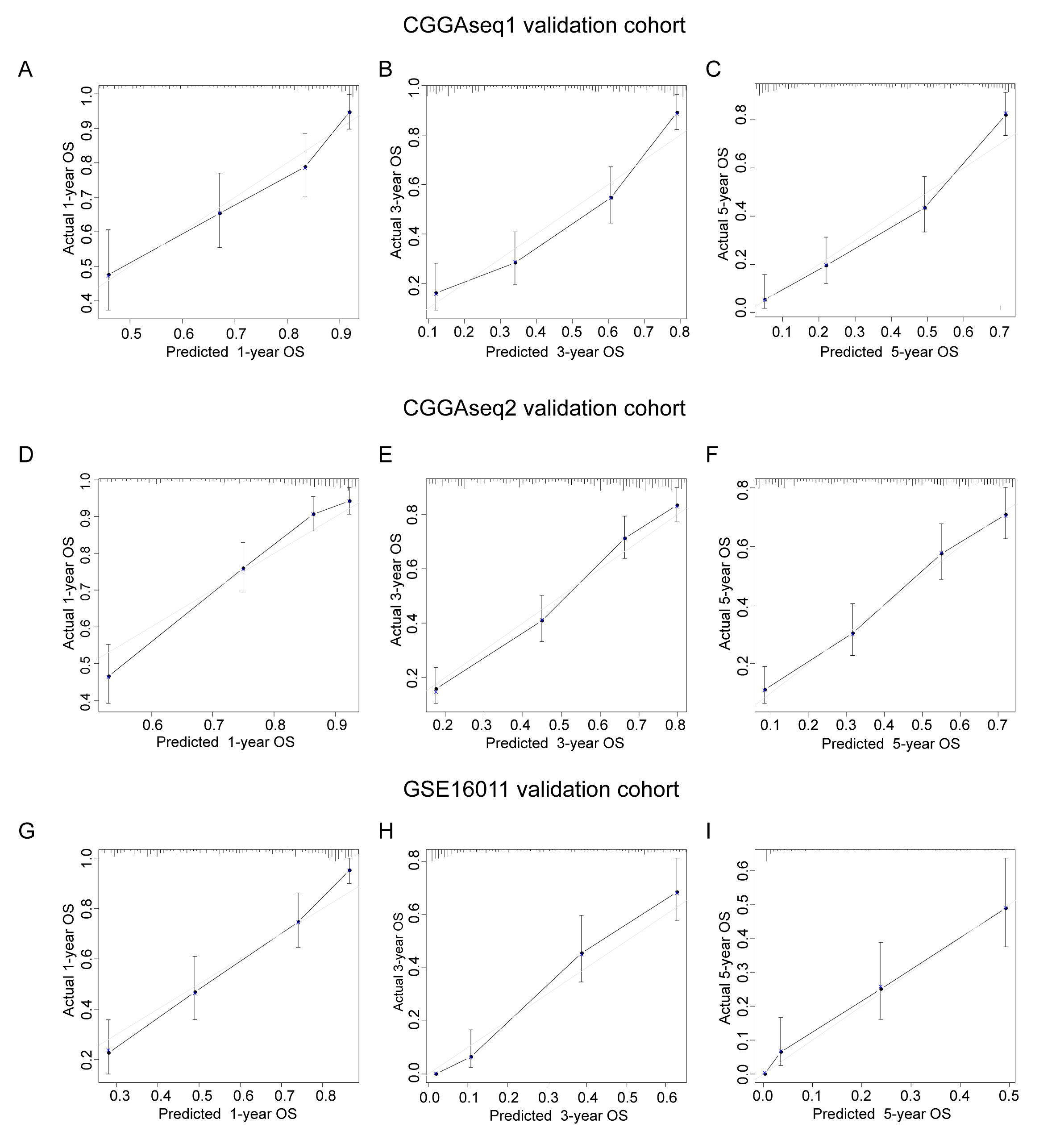

Supplement: Supplementary file 3 [file Image_3.TIF]
